# Cardiogenic shock in Taiwan from 2003 to 2017 (CSiT-15 study)

**DOI:** 10.1186/s13054-021-03820-1

**Published:** 2021-11-18

**Authors:** Shih-Chieh Chien, Chien-Yi Hsu, Hung-Yi Liu, Chao-Feng Lin, Chung-Lieh Hung, Chun-Yao Huang, Li-Nien Chien

**Affiliations:** 1 Department of Critical Care Medicine, MacKay Memorial Hospital, Taipei, Taiwan; 2 Cardiovascular Division, Department of Internal Medicine, MacKay Memorial Hospital, Taipei, Taiwan; 3 Division of Cardiology and Cardiovascular Research Center, Department of Internal Medicine, Taipei Medical University Hospital, Taipei, Taiwan; 4 Division of Cardiology, Department of Internal Medicine, School of Medicine, College of Medicine, Taipei Heart Institute, Taipei Medical University, Taipei, Taiwan; 5 Health Data Analytics and Statistics Center, Office of Data Science, Taipei Medical University, No. 250 Wuxing Street, Taipei, Taiwan; 6 School of Health Care Administration, College of Management, Taipei Medical University, Taipei, Taiwan

**Keywords:** Cardiogenic shock, Mortality, Medical costs, Acute myocardial infarction, Intra-aortic balloon pump, Policy, Taiwan

## Abstract

**Background:**

This study investigated temporal trends in the treatment and mortality of patients with cardiogenic shock (CS) in Taiwan in relation to acute myocardial infarction (AMI) accreditation implemented in 2009 and the unavailability of percutaneous ventricular assist devices.

**Methods:**

Data of patients diagnosed as having CS between January 2003 and December 2017 were collected from Taiwan’s National Health Insurance Research Database. Each case was followed from the date of emergency department arrival or hospital admission for the first incident associated with a CS diagnosis up to a 1-year interval. Measurements included demographics, comorbidities, treatment, mortality, and medical costs. Using an interrupted time-series (ITS) design with multi-level mixed-effects logistic regression model, we assessed the impact of AMI accreditation implementation on the mortality of patients with AMI and CS overall and stratified by the hospital levels.

**Results:**

In total, 64 049 patients with CS (mean age:70 years; 62% men) were identified. The incidence rate per 10^5^ person-years increased from 17 in 2003 to 25 in 2010 and plateaued thereafter. Average inpatient costs increased from 159 125 points in 2003 to 240 993 points in 2017, indicating a 1.5-fold increase. The intra-aortic balloon pump application rate was approximately 22–25% after 2010 (*p* = 0.093). Overall, in-hospital, 30-day, and 1-year mortality declined from 60.3%, 63.0%, and 69.3% in 2003 to 47.9%, 50.8% and 59.8% in 2017, respectively. The decline in mortality was more apparent in patients with AMI-CS than in patients with non-AMI-CS. The ITS estimation revealed a 2% lower in-hospital mortality in patients with AMI-CS treated in district hospitals after the AMI accreditation had been implemented for 2 years.

**Conclusions:**

In Taiwan, the burden of CS has consistently increased due to high patient complexity, advanced therapies, and stable incidence. Mortality declined over time, particularly in patients with AMI-CS, which may be attributable to advancements in AMI therapies and this quality-improving policy.

**Supplementary Information:**

The online version contains supplementary material available at 10.1186/s13054-021-03820-1.

## Introduction

The clinical features of cardiogenic shock (CS) have considerably changed over the past two decades because of improved knowledge regarding its pathophysiological mechanisms, therapeutic advancements, and collaborative care [1–6]. In-hospital mortality declined from 80 to 30–50% [1, 5, 7–10], although some studies have observed a small rebound [5, 8]. Although patients presented with a higher number of comorbidities and coronary lesions, the proportion of patients with acute myocardial infarction–associated CS (AMI-CS) decreased [5, 8]. Furthermore, the use of intra-aortic balloon pumps (IABPs) decreased, whereas that of percutaneous ventricular assist devices (VADs) increased [1]. While there are studies reported recent outcomes in CS, those evidences are mostly generated from acute myocardial infarction (AMI)–based studies. Evidence for the non-AMI population remains insufficient [1, 5, 7–10].

The government has implemented accreditation program for AMI to improve quality of care since 2009 in Taiwan. The impact of accreditation program on outcomes in CS patients has not been evaluated. Since percutaneous VADs including Impella (Abiomed Europe, Aachen, Germany) and TandemHeart (Cardiac Assist, Inc, Pittsburgh, PA, USA) are not available in Taiwan, the impact of inaccessibility of such devices on clinical outcomes is not known. Therefore, we conducted a nationwide longitudinal cohort study by using real-world data to investigate temporal trends in the incidence, medical costs, mechanical circulatory support (MCS) devices, and mortality of CS in Taiwan over the past 15 years. The findings of this study can improve our understanding regarding the features of CS with heterogenous etiologies.

## Material and methods

### Data source

The universal compulsory National Health Insurance (NHI) program was launched by the Taiwanese government in March 1995. Taiwan’s National Health Insurance Research Database (NHIRD) contains complete information regarding outpatient and emergency visits, hospital admissions, medication prescriptions, disease diagnoses, medical procedures, and vital statuses for 99% of Taiwan’s population (~ 23 million). Diagnoses in the NHIRD are coded according to the *International Classification of Diseases, Ninth Revision, Clinical Modification* (*ICD-9-CM*) and *International Classification of Diseases, Tenth Revision, Clinical Modification* (*ICD-10-CM*) since 2016. The NHIRD can be linked with the National Death Registry (NDR) by using the unique encrypted identification number of each beneficiary. The accuracy of data in the NHIRD and NDR has been analyzed in previous studies [11, 12]. The study protocol was approved by the Joint Institutional Review Board of Taipei Medical University (approval no.: N202012062).

### Interrupted time-series study design

To examine the effect of AMI accreditation on the mortality of patients with AMI-CS, we adopted an interrupted time-series (ITS) study design. In brief, ITS is a robust quasi-experimental design in which randomized controlled trials cannot be used. In an ITS design, data are collected at multiple and equally spaced time points (in this case, yearly) before and after intervention (in this case, AMI accreditation). The main objective of an ITS is to examine changes in the data pattern pre- and post-intervention [13]. In this study, the pre- and post-intervention periods were from 2003 to 2008 and from 2009 to 2017, respectively. In addition, we hypothesized that AMI accreditation would exert a lag effect on mortality and that hospitals of different levels would be differentially affected by the policy. Therefore, we investigated the lag effect of the AMI accreditation policy and performed a subgroup analysis by the hospital level (medical centers, regional hospitals, and district hospitals).

### Study population

From the NHIRD, we identified patients who received a diagnosis of CS (ICD-9-CM code 785.51 or ICD-10-CM code R570) in an inpatient or emergency department between January 1, 2003, and December 31, 2017. We excluded patients who (1) had missing age or sex information (*n* = 213), (2) were younger than 18 years (*n* = 687), and (3) had a length of stay of more than 365 days (*n* = 14). The last exclusion criterion was added because primary endpoints could not be evaluated. Each case was followed from the date of emergency department arrival or hospital admission for the patients’ first incident associated with a CS diagnosis up to a 1-year interval.

### National accreditation policy associated with AMI

The program of hospital accreditation for emergency ability was implemented in 2009 to integrate emergency network systems and provide timely high-quality emergency care under the Emergency Medical Services Act [14, 15]. The central health authorities, namely the Joint Commission of Taiwan and the Ministry of Health and Welfare, are responsible for certifying the accreditation of emergency medical capability that is classified as severe-, moderate-, and general-level every 2 to 4 years. In addition, local health authorities must ensure that accredited hospitals meet their requirements annually. Severe-level hospitals are specifically designated as the last-line hospital referral for those who require specific mechanical support or advanced therapies. Although only medical centers are designated as severe-level hospitals, regional hospitals can also apply and be accredited as severe-level hospitals through the same evaluation process. The annual hospital numbers of different levels are presented in Additional file 1.

For AMI, severe-level hospitals are required to provide 24-h medical service; however, moderate-level hospitals may provide a daytime or specific-hour service. Medical services for patients with AMI, particularly those with ST-segment elevation myocardial infarction (STEMI), are recorded and reviewed by the central or local health authorities for the level-specific quality assessment. For example, at least 80% of patients with STEMI must receive initial electrocardiography within 10 min, dual-antiplatelet and fibrinolytic therapies within 30 min, or percutaneous coronary intervention (PCI) within 90 min as required in severe-level hospitals [14]. Moreover, data regarding beta blocker prescriptions for AMI before discharge has been included in the accreditation since 2015. All emergency medical technicians are fully trained to respond to the situation and must directly transport patients suspected of having AMI to a certified hospital nearby for evaluation of the need of immediate revascularization.

### Mortality

Outcomes were defined as short-term (in-hospital and 30-day) and long-term (1-year) mortality; these data were obtained from either the NHIRD or NDR. Patients were considered to have died in the hospital if their death record was issued in the hospital or within 2 days after the date of hospital discharge.

### Covariates

The selected covariates in the study were thought fundamentally or prognostically important for patients with CS, namely hypertension, dyslipidemia, coronary artery disease, prior myocardial infarction, renal failure, congestive heart failure, peripheral arterial disease, and atrial fibrillation [5, 7–10]. Patients were considered to have a specific disease if they had at least two diagnostic claims during outpatient visits or one diagnostic claim during hospital admission 1 year prior to the index date of CS. Medical procedures performed during CS admission were recorded and included PCI; coronary artery bypass grafting (CABG); heart transplantation; inotrope or vasopressor therapy using dopamine, norepinephrine, dobutamine, and epinephrine; and MCS with IABPs, extracorporeal membrane oxygenation (ECMO), and VADs. Diagnostic disease codes, medication use, and treatment procedures are detailed in Additional file 2.

### Statistical analysis

We presented individual-level data for demographics, incidence, healthcare resource use, medical costs, and mortality of patients with CS across different time periods. The chi-squared test for linear trends was employed to examine changes of baseline over time. A multi-level mixed-effects logistic regression model that has a random intercept for center/hospital of care was used to examine the policy effect on in-hospital mortality before (2003–2008) and after (2009–2017) the introduction of AMI accreditation [16]. Also, we reported the effect of AMI accreditation based on predicted probabilities calculated from the models. Marginal standardization method was chosen because it was suggested to adopt when making inference to the overall population and the standard error estimation was calculated using the delta method [17]. In the NHIRD, the cost data are reported as points. Since we use global budget payment system in Taiwan, the conversion ratio of points to Taiwan dollars varies every year but close to one. Therefore, “points” reflects complexity of care more accurately than “dollars” in Taiwan. For better understanding, costs were also converted to US dollars by using the 2017 exchange rate (1:30). Statistical analyses were conducted using SAS/STAT v9.4 (SAS Institute, Cary, NC, USA) and STATA/SE 16 (StataCorp, College Station, TX, USA). A *p*  < 0.05 was considered statistically significant.

## Results

### Incidence, costs, and length of hospital stay

**Fig. 1:**
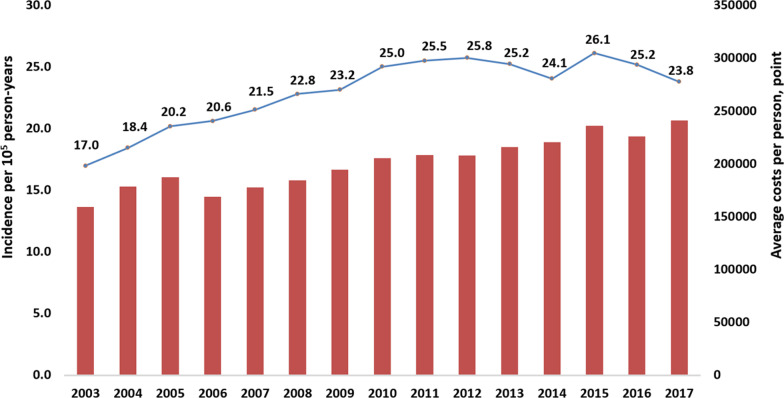
Annual incidence per 10^5^ person-years and average medical costs of cardiogenic shock. The crude incidence increased from 2003 to 2010 and stabilized after 2010. Medical costs consistently increased over time

From January 2003 to December 2017, a total of 64 963 CS cases were identified; of these, 64 049 fulfilled the inclusion criteria. The crude incidence rate per 10^5^ person-years increased from 17 in 2003 to 25 in 2010 and plateaued thereafter, with annual numbers ranging from 4500 to 5100. In addition, average inpatient costs increased from 159 125 points in 2003 to 240 993 points in 2017, approximately US$5304 in 2003 to US$8033 in 2017 (Fig. 1; Additional file 3). Both the average length of stay in the intensive care unit and hospital slightly increased over the entire period (Additional file 3).

### Baseline characteristics and treatment

Table 1 lists baseline characteristics across time periods. Overall, the mean age of patients was 70 years, and more than 60% of patients were men. The prevalence of comorbidities and cardiovascular diseases increased over time; however, the risk of stroke decreased. Compared with earlier study periods, patients in the later period (2015–2017) were more likely to be diagnosed as having AMI (~ 40%), treated at a medical center or regional hospital, and receive PCI and norepinephrine therapy; however, they were less likely to have experienced cardiac arrest and receive CABG, dopamine, dobutamine, or epinephrine therapy. Generally, the percentage of MCS device use was between 20 and 27% (Additional file 4). The IABP was the most commonly used device, with estimated use ranging from 17.2 to 23.4% (*p* for trend after 2010 = 0.093). Approximately 10% of patients with CS received ECMO therapy, surgical but not percutaneous VADs were implemented sporadically, with approximately 20 cases per year reported after 2012. Ultimately, only 0.3% of patients with CS received heart transplantation. Baseline data stratified by AMI etiology and 1-year mortality are also presented in Additional files 5 and 6.

**Table 1:** Baseline characteristics of patients with cardiogenic shock stratified by different time periods

	Overall	2003–2005	2006–2008	2009–2011	2012–2014	2015–2017	Trend *p*
	*N* = 64 049	*N* = 9657	*N* = 11 677	*N* = 13 686	*N* = 14 342	*N* = 14 687	
*Demographics*							
Age (years)	70.6 ± 14.8	70.3 ± 14.0	70.5 ± 14.5	70.9 ± 14.7	70.9 ± 15.2	70.3 ± 15.2	< 0.0001
Male sex (%)	39 706 (62%)	5887 (61%)	7141 (61.2%)	8448 (61.7%)	8969 (62.5%)	9261 (63.1%)	< 0.0001
*History, n (%)*							
Congestive heart failure	26 310 (41.1%)	3588 (37.2%)	4553 (39%)	5747 (42%)	6138 (42.8%)	6284 (42.8%)	< 0.0001
Hypertension	30 941 (48.3%)	4025 (41.7%)	5318 (45.5%)	6760 (49.4%)	7349 (51.2%)	7489 (51%)	< 0.0001
Diabetes mellitus	22 503 (35.1%)	3153 (32.6%)	4089 (35%)	5005 (36.6%)	5133 (35.8%)	5123 (34.9%)	0.001
Peripheral arterial disease	780 (1.2%)	69 (0.7%)	112 (1%)	143 (1%)	202 (1.4%)	254 (1.7%)	< 0.0001
Dyslipidemia	9604 (15%)	942 (9.8%)	1476 (12.6%)	1918 (14%)	2389 (16.7%)	2897 (19.6%)	< 0.0001
Coronary artery disease	29 960 (46.8%)	4229 (43.8%)	5208 (44.6%)	6272 (45.8%)	6973 (48.6%)	7278 (49.6%)	< 0.0001
Prior myocardial infarction	28 885 (45.1%)	4117 (43.3%)	5049 (43.2%)	6233 (45.5%)	6551 (45.7%)	6875 (46.8%)	< 0.0001
Renal failure	5561 (8.7%)	550 (5.7%)	858 (7.3%)	1170 (8.5%)	1431 (10%)	1552 (10.6%)	< 0.0001
Stroke	11 091 (17.3%)	1749 (18.1%)	2217 (19%)	2439 (17.8%)	2485 (17.3%)	2201 (15%)	< 0.0001
Malignancy	6214 (9.7%)	850 (8.8%)	1099 (9.4%)	1336 (9.8%)	1479 (10.3%)	1450 (9.9%)	0.0004
Atrial fibrillation	9490 (14.8%)	1279 (13.2%)	1747 (15%)	2024 (14.8%)	2183 (15.2%)	2257 (15.4%)	< 0.0001
*Hospital level, n (%)*							
Medical center	22 499 (35.1%)	3359 (34.8%)	4022 (34.4%)	4960 (36.2%)	5019 (35%)	5139 (35%)	0.3001
Regional hospital	32 052 (50%)	4420 (45.8%)	5702 (48.8%)	6790 (49.6%)	7420 (51.7%)	7720 (52.6%)	< 0.0001
District hospital	9498 (14.8%)	1878 (19.4%)	1953 (16.7%)	1936 (14.1%)	1903 (13.3%)	1828 (12.4%)	< 0.0001
*CS conditions, n (%)*							
Cardiac arrest	21 095 (32.9%)	3525 (36.5%)	4222 (36.2%)	4597 (33.6%)	4479 (31.2%)	4272 (29.1%)	< 0.0001
Acute myocardial infarction	25 906 (40.4%)	3757 (38.9%)	4524 (38.7%)	5580 (40.8%)	5805 (40.5%)	6240 (42.5%)	< 0.0001
STEMI	9522 (14.9%)	1808 (18.7%)	1726 (14.8%)	1964 (14.4%)	1906 (13.3%)	2118 (14.4%)	< 0.0001
NSTEMI	16 384 (25.6%)	1949 (20.2%)	2798 (24%)	3616 (26.4%)	3899 (27.2%)	4122 (28.1%)	< 0.0001
*Cardiac procedure, n (%)*							
PCI	15 465 (24.1%)	1447 (15%)	2205 (18.9%)	3248 (23.7%)	3878 (27%)	4687 (31.9%)	< 0.0001
CABG	3653 (5.7%)	828 (8.6%)	782 (6.7%)	745 (5.4%)	707 (4.9%)	591 (4%)	< 0.0001
Heart transplantation	183 (0.3%)	20 (0.2%)	29 (0.2%)	47 (0.3%)	44 (0.3%)	43 (0.3%)	0.0921
*Vasoactive agents, n (%)*							
Dopamine	48 155 (75.2%)	7668 (79.4%)	9289 (79.5%)	10,751 (78.6%)	10,563 (73.7%)	9884 (67.3%)	< 0.0001
Norepinephrine	24 817 (38.7%)	2563 (26.5%)	3929 (33.6%)	5096 (37.2%)	6220 (43.4%)	7009 (47.7%)	< 0.0001
Dobutamine	12 123 (18.9%)	2720 (28.2%)	2665 (22.8%)	2592 (18.9%)	2230 (15.5%)	1916 (13%)	< 0.0001
Epinephrine	34 806 (54.3%)	5815 (60.2%)	6703 (57.4%)	7724 (56.4%)	7500 (52.3%)	7064 (48.1%)	< 0.0001
*Mechanical support, n (%)*							
IABP	13 782 (21.5%)	1835 (19%)	2413 (20.7%)	3172 (23.2%)	3003 (20.9%)	3359 (22.9%)	< 0.0001
ECMO	5915 (9.2%)	993 (10.3%)	1202 (10.3%)	1103 (8.1%)	1247 (8.7%)	1370 (9.3%)	< 0.0001
VAD	140 (0.2%)	0	0	15 (0.1%)	58 (0.4%)	67 (0.5%)	< 0.0001

CS, cardiogenic shock; STEMI, ST-segment elevation myocardial infarction; NSTEMI, non-ST-segment elevation myocardial infarction; PCI, percutaneous coronary intervention; CABG, coronary artery bypass graft; IABP, intra-aortic balloon pump; ECMO, extracorporeal membrane oxygenation; VAD, ventricular assist device

### Mortality

**Fig. 2:**
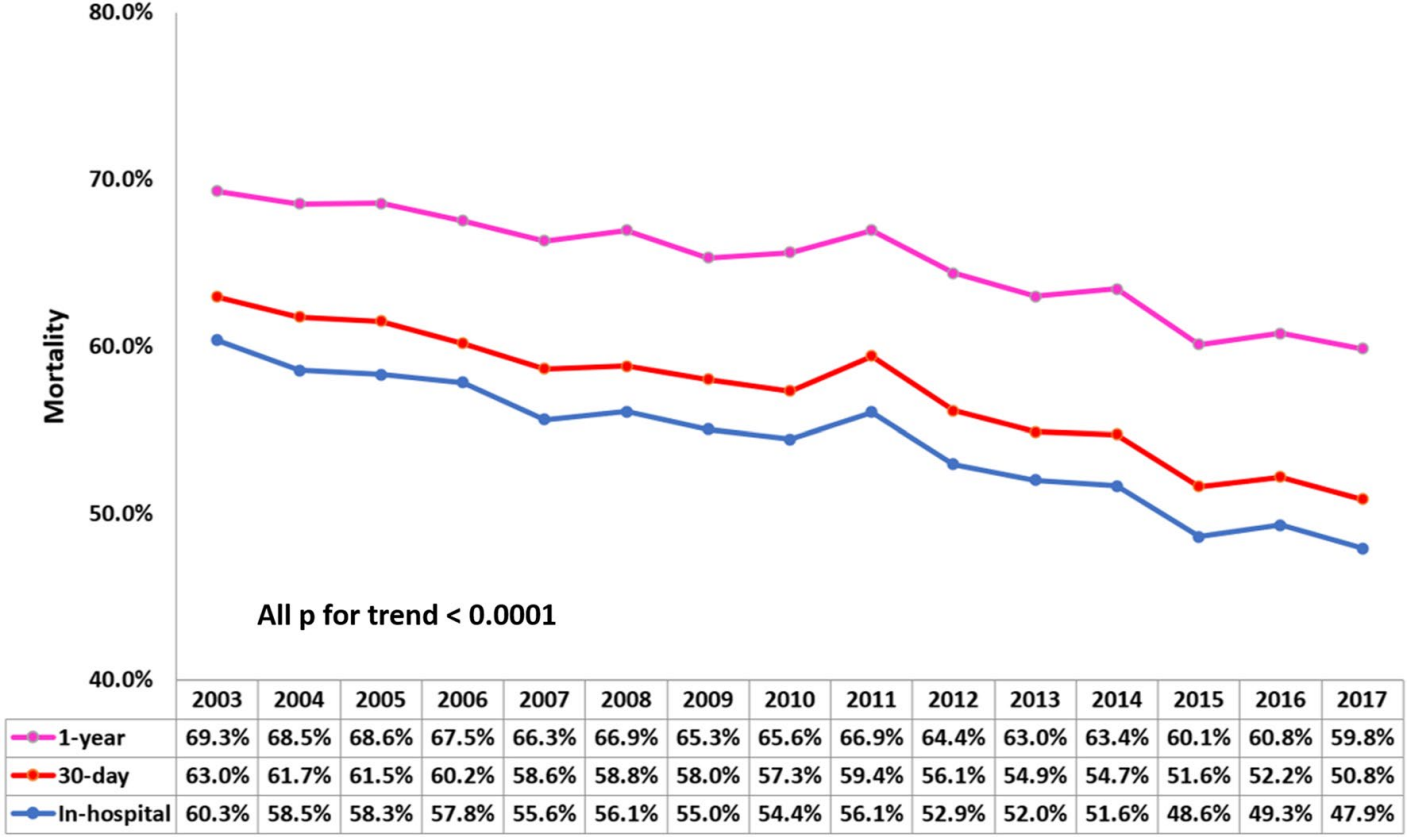
Annual in-hospital, 30-day, and 1-year mortality rate. The unadjusted in-hospital, 30-day, and 1-year mortality rates consistently declined from 60.3%, 63%, and 69.3% in 2003 to 47.9%, 50.8%, and 59.8% in 2017, respectively

**Fig. 3:**
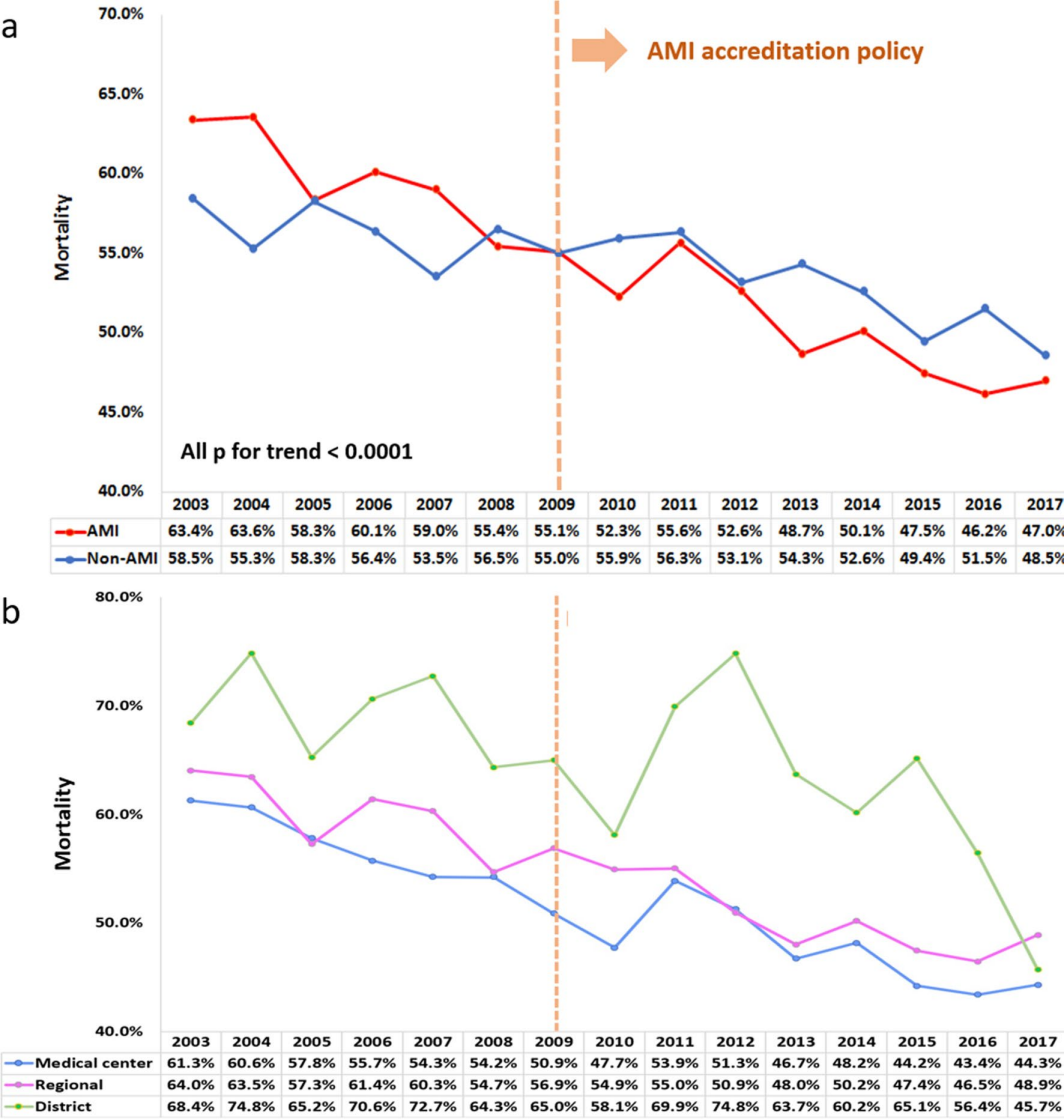
**a** The unadjusted annual in-hospital mortality stratified by AMI etiology and **b** hospital level of the AMI-CS population. **a** Declines in annual mortality were observed in both groups. **b** The in-hospital mortality rate was the highest in patients treated in district hospitals and the lowest in those treated in medical centers. However, these differences decreased in the later period. AMI, acute myocardial infarction; CS, cardiogenic shock

The unadjusted data showed that in-hospital, 30-day, and 1-year mortality declined from 60.3%, 63%, and 69.3% to 47.9%, 50.8%, and 59.8%, respectively, gradually decreasing over the entire study period (Fig. 2). Moreover, the unadjusted in-hospital mortality of patients with AMI and non-AMI exhibited a decreasing temporal trend during the follow-up period (Fig. 3a). Notably, mortality risk for AMI-CS was higher compared with that for non-AMI-CS before 2009, but it became lower after 2009. For patients with AMI-CS (Fig. 3b), we observed that in-hospital mortality was the highest when they were treated in district hospitals and the lowest when treated in medical centers; however, these differences decreased in the later period.

### Marginal effect of AMI accreditation

The ITS estimation (detailed in Table 2) represented the marginal effect of AMI accreditation on in-hospital mortality. Overall, AMI accreditation had no effect on in-hospital mortality (0.05%, 95% confidence interval [CI]: − 0.002 to 0.012, *p* = 0.171). In terms of the effect of AMI accreditation involving longer periods of time, the association of the policy with mortality remained nonsignificant. When analyses were stratified by the hospital level, a 2.0% lower in mortality (95% CI of − 3.9% to − 0.01%, *p* = 0.041) was observed in patients treated in district hospitals after the AMI accreditation had been implemented for 2 years. Moreover, the effect continued into later years (− 2.9% in 2012; − 2.6% in 2013).

**Table 2:** The marginal effect of AMI accreditation on in-hospital mortality of AMI-CS patients, by level of hospital

	Overall			Medical center			Regional hospital			District hospital		
	dy/dx	(95% CI)	*P*	dy/dx	(95% CI)	*P*	dy/dx	(95% CI)	*P*	dy/dx	(95% CI)	*P*
*Policy: 2009*												
t	− 0.016	(− 0.022, − 0.010)	< .0001*	− 0.007	(− 0.012, − 0.002)	0.004*	− 0.021	(− 0.031, − 0.012)	< .0001*	− 0.006	(− 0.025, 0.013)	0.514
P_t_	0.005	(− 0.019, 0.028)	0.688	− 0.003	(− 0.021, 0.015)	0.708	0.012	(− 0.021, 0.045)	0.477	0.024	(− 0.054, 0.101)	0.549
[*t* − *T*_*I*_ ]P_*t*_	0.005	(− 0.002, 0.012)	0.171	0.002	(− 0.003, 0.008)	0.344	0.009	(− 0.001, 0.019)	0.078	− 0.007	(− 0.028, 0.014)	0.519
*Policy lag: 2010*												
t	− 0.015	(− 0.020, − 0.010)	< .0001*	− 0.008	(− 0.012, − 0.004)	< .0001*	− 0.017	(− 0.025, − 0.010)	< .0001*	− 0.008	(− 0.023, 0.008)	0.323
P_t_	0.006	(− 0.017, 0.029)	0.612	0.004	(− 0.013, 0.022)	0.624	0.002	(− 0.030, 0.035)	0.897	0.040	(− 0.039, 0.118)	0.321
[*t* − *T*_*I*_]P_*t*_	0.004	(− 0.002, 0.009)	0.224	0.003	(− 0.002, 0.007)	0.234	0.006	(− 0.003, 0.015)	0.164	− 0.008	(− 0.027, 0.011)	0.385
*Policy lag: 2011*												
t	− 0.016	(− 0.020, − 0.012)	< .0001*	− 0.008	(− 0.012, − 0.005)	< .0001*	− 0.016	(− 0.022, − 0.010)	< .0001*	− 0.010	(− 0.023, 0.003)	0.120
D_t_	0.023	(0.000, 0.046)	0.051	0.021	(0.004, 0.038)	0.016	− 0.007	(− 0.040, 0.026)	0.676	0.110	(0.031–0.189)	0.006
[*t* − * T*_*I*_]P_*t*_	0.002	(− 0.004, − 0.008)	0.489	0.001	(− 0.003, 0.005)	0.624	0.006	(− 0.002, 0.014)	0.165	− 0.020	(− 0.039, − 0.001)	0.041*
*Policy lag: 2012*												
t	− 0.013	(− 0.016, 0.010)	< .0001*	− 0.007	(− 0.010, 0.004)	< .0001*	− 0.014	(− 0.019, − 0.010)	< .0001*	− 0.005	(− 0.015, 0.006)	0.376
D_t_	0.005	(− 0.018, 0.028)	0.678	0.009	(− 0.010, 0.028)	0.364	− 0.015	(− 0.047, 0.018)	0.382	0.088	(0.006, 0.171)	0.035*
[*t* − *T*_*I*_]P_*t*_	0.003	(− 0.003, 0.009)	0.292	0.001	(− 0.003, 0.006)	0.559	0.008	(− 0.000, 0.016)	0.056	− 0.029	(− 0.051, 0.008)	0.006*
*Policy lag: 2013*												
t	− 0.012	(− 0.015, − 0.009)	< .0001*	− 0.007	(− 0.009, 0.004)	< .0001*	− 0.014	(− 0.018, − 0.010)	< .0001*	− 0.010	(− 0.023, 0.003)	0.120
D_t_	− 0.009	(− 0.034, 0.016)	0.473	0.004	(− 0.017, 0.024)	0.723	− 0.022	(− 0.058, 0.014)	0.224	0.110	(0.031–0.189)	0.006
[*t* − *T*_*I*_]P_*t*_	0.005	(− 0.002, 0.012)	0.174	0.001	(− 0.005, 0.007)	0.677	0.010	(− 0.001, 0.020)	0.054	− 0.026	(− 0.051, 0.001)	0.040*

Time variable referred as t was beginning at 1 in the year of 2002 and incrementing by 1 up to the year of 2017; policy variable referred as P_t_ (an intervention time indicator) was coded 0 pre-intervention and 1 post-intervention; the marginal effect of the policy referred as [*t* − *T*_*I*_]P_*t*_ (a slope change variable) was equal to zero at the time of the intervention (*T*_*I*_) and incrementing by 1 up to the year of 2017. dx/dy referred as marginal effect was estimated by using multi-level mixed effect logistic regression models which had a random intercept for center and adjusted for individual-level demographics and disease history listed in Table 1

*P < 0.05

## Discussion

In this large population-based study, we used real-world data to determine several critical issues related to CS in Taiwan. First, the burden of CS was substantial and continued to grow despite the incidence remaining stable. Second, AMI contributed to approximately 40% of all instances of CS, and patients presented with more cardiovascular comorbidities. Third, trends for revascularization and inotrope or vasopressor therapy changed over the study period, whereas the application of IABPs generally remained stable after 2010. Finally, the survival of patients with CS continually improved; the decline in mortality was more predominant in patients with AMI-CS than in those with non-AMI-CS, possibly owing to therapeutic advances and this quality-improving policy.

### Trends in incidence, medical costs, and patient characteristics

Contemporary epidemiological studies on CS have mainly focused on populations with AMI, with incidence trends for overall CS being less frequently reported [5]. The data from the 15-year period in this study indicated a CS incidence rate of 15–30 per 10^5^ person-years, which was slightly higher than that reported in the United States [5]. In Taiwan, the increasing incidence of CS from 2003 to 2010 may reflect a decrease in underdiagnosis and inappropriate coding. The treatment of a patient with CS typically requires intensive resource use [5, 7, 18, 19], which resulted in a 1.5-fold increase in medical costs from 2003 to 2017 following the introduction of innovative devices and advanced treatments. The continual increase in the numbers and severity of patients with CS would have significantly increased the financial burden on Taiwan’s NHI program [18]. In addition, patients with CS presented with more cardiovascular comorbidities in recent years; this finding is consistent with that of a previous study [8]. However, fewer patients with CS experienced cardiac arrest; this result is inconsistent with those of other studies [5, 20]. Trends in PCI increased from 15 to 31.9%, but those in CABG decreased from 8.6 to 4% between 2003 and 2017. Moreover, such a rapid rise in the rate of PCI has been observed in other cohorts and registries [10, 20]

### Trends in vasoactive agents and MCS

Norepinephrine is the first-line medication for increasing blood and tissue perfusion pressure in patients with CS rather than dopamine or epinephrine [2, 21, 22]. The pharmacological trends for patients in our study are consistent with current recommendations. Our results exhibited a decrease in dobutamine use over time. In our study, IABPs were the most commonly used device (~ 22–25%). In contrast to other studies, we did not observe a continually declining rate of IABP use following the publication of the Intra-Aortic Balloon Pump in Cardiogenic Shock II trial [5, 8, 20]. One possible explanation was the inaccessibility of alternative percutaneous VADs, such as Impella and TandemHeart, in Taiwan. Moreover, IABPs can be implemented more readily compared with other MCS devices and provide modest hemodynamic benefits [23–25]. Although ECMO can provide more comprehensive cardiopulmonary support than an IABP, the clinical application of ECMO remains limited because of the complex circuit preparation, patient care troubleshooting, and high complication rate [26–28]. Although different MCS devices have various advantages and disadvantages, all MCS devices must be made available in CS centers for intensivists or cardiologists to select the appropriate method to meet the patients’ needs [29].

### Policy implementation: effect and mortality

We observed a continual decline in short- and long-term mortality in patients with CS in Taiwan. The in-hospital mortality rate of 48% in 2017 is similar to but lower than that reported by the US National Institutes of Health [5] but higher than those reported from other registries or AMI cohorts [8, 20, 30, 31]. Notably, our data indicated that more than one in five hospital survivors would die within 1 year following the index date of CS; thus, the integration of post-discharge management in this high-risk population is warranted.

Although AMI was considered a risk factor for mortality in CS [9], substantial studies, including our study, have reappraised its prognostic role [6]. Compared with non-AMI-CS, more evidence-based medications and interventions have been developed to target AMI-CS in the last two decades [1, 2]. A more predominant improvement of survival in patients with AMI-CS has been achieved through the reinforcement of guideline-directed therapies than in those with non-AMI-CS.

A lower in-hospital mortality in district hospitals implied that the direct implementation of AMI accreditation enhanced the structure and process of care in district hospitals but had little prognostic effect in relatively high-quality and well-equipped hospitals. This finding suggests that the AMI accreditation policy helped raising public awareness, facilitating prehospital patient transportation, and establishing a hospital referral system. The decreasing trend of patients with CS treated in district hospitals may support this putative interpretation. This result constitutes vital information for policymaking or policy modifications in the future.

However, an overemphasis on AMI-relevant aspects might have resulted in imbalanced medical resources as well as inadequate treatment in patients with non-AMI-CS. This was reflected in the markedly lower uses of IABPs and ECMO in the non-AMI-CS group compared with the AMI-CS group (IABP: 9% vs 40%; ECMO: 7.1% vs 12.4%, respectively) in our study. Healthcare authorities must draw more attention to the treatment of non-AMI-CS and prevent medical futility in AMI-CS treatment.

### Study limitations

Our study has several limitations. First, the current study was an observational retrospective claim-based study in the real-world settings. Because clinically relevant imaging and laboratory data were unavailable, the validity of CS diagnosis was mainly dependent on discharge claims. Second, eliminating disease heterogeneity (e.g., disease severity) in our study population was difficult and may have affected our finding in relation to the effect of AMI accreditation. Third, we were unable to study medications and devices not covered by the NHI program (e.g., levosimendan). Fourth, we recognized that the impact of AMI accreditation would be weakened since we were not able to directly compare the mortality of patients treated or not treated in accredited hospitals. Finally, other AMI-relevant interventions might affect the in-hospital mortality of AMI-CS patients. Since none of those interventions were implemented nationwide, the impact was likely to be small.

## Conclusions

The burden of CS has consistently increased in Taiwan because of high patient complexity, advanced therapies, and stable incidence. Without available percutaneous VADs, IABP utilization rate remained unchanged (20–24%). The in-hospital mortality rate decreased from 60% in 2003 to < 50% between 2015 and 2017, but 1-year mortality remained high (60%). In particular, a lower mortality risk was observed in the AMI-CS group than that in the non-AMI-CS group after 2009, possibly reflecting advancements in AMI therapies and the quality-improving policy. Promotion of the post-acute care in patients with CS and therapeutic interventions focusing on patients with non-AMI-CS are essential.

## Supplementary Information

**Additional file 1.** Annual hospital number according to their accredited level for emergency medical ability.

**Additional file 2.** Disease diagnostic coding, procedure coding, and ATC code for medication.

**Additional file 3.** Healthcare resource use stratified by different time periods.

**Additional file 4.** Annual trends of mechanicals circulatory support devices.

**Additional file 5.** Baseline characteristics of patients with cardiogenic shock stratified by AMI etiology.

**Additional file 6.** Baseline characteristics of patients with cardiogenic shock stratified by 1-year mortality. 

## Data Availability

With regard to data availability, our study used healthcare administrative data provided by the third-party organization Health and Welfare Science Data Center (HWDC), Ministry of Health and Welfare in Taiwan. Researchers can submit applications to the HWDC to gain access to several health-related databases. Because of legal restrictions imposed by the government of Taiwan in relation to the Personal Information Protection Act, data cannot be made publicly available. Requests for data can be sent as formal proposals to the HWDC with IRB approval stating research purposes only. Taipei Medical University’s Joint IRB can be contacted at tmujirb@gmail.com. All data were fully anonymized before we accessed and analyzed them in an independent operating area in the HWDC. Only statistical results can be removed from the operating area. Therefore, original data cannot be shared publicly because of legal restrictions involved.

## Abbreviations

CS: Cardiogenic shock
AMI: Acute myocardial infarction
AMI-CS: Acute myocardial infarction-associated cardiogenic shock
IABP: Intra-aortic balloon pump
VAD: Ventricular assist device
MCS: Mechanical circulatory support
NHI: National Health Insurance
NHIRD: National Health Insurance Research Database
ICD-9-CM: International Classification of Diseases, Ninth Revision, Clinical Modification
ICD-10-CM: International Classification of Diseases, Tenth Revision, Clinical Modification
NDR: National Death Registry
STEMI: ST-segment elevation myocardial infarction
NSTEMI: Non-ST-segment elevation myocardial infarction
PCI: Percutaneous coronary intervention
CABG: Coronary artery bypass graft
ECMO: Extracorporeal membrane oxygenation
ITS: Interrupted time-series
