# Supplementary material for: Cardiogenic shock in Taiwan from 2003 to 2017 (CSiT-15 study)
Source: Crit Care. 2021 Nov 18;25:402. doi: 10.1186/s13054-021-03820-1 (PMC8600726; doi:10.1186/s13054-021-03820-1)
Supplement: Supplementary file 1 — Additional file 1. Annual hospital number according to their accredited level for emergency medical ability. [file 13054_2021_3820_MOESM1_ESM.doc]

**Additional file 1**. Annual hospital number according to their accredited level for emergency medical ability

Description of data: This table describes annual hospital numbers according to their accredited emergency level.

|  | 2009 | 2010 | 2011 | 2012 | 2013 | 2014 | 2015 | 2016 | 2017 |
| --- | --- | --- | --- | --- | --- | --- | --- | --- | --- |
| Severe, n (%) | 16 (8) | 23 (11.4) | 26 (13.8) | 27 (14.4) | 31 (15.5) | 34 (17.8) | 36 (18.6) | 39 (20) | 39 (19.7) |
| Moderate, n (%) | 18 (9) | 33 (16.4) | 66 (34.9) | 76 (40.6) | 82 (41) | 79 (41.4) | 82 (42.3) | 80 (41) | 81 (40.9) |
| General, n (%) | 165 (82.9) | 145 (72.1) | 97 (51.3) | 84 (44.9) | 87 (43.5) | 78 (40.8) | 76 (39.2) | 76 (39) | 78 (39.4) |

Hospitals which did not provide emergency medical services were not included.

Data were confirmed with the Department of Medical Affairs of Taiwan’s Ministry of Health and Welfare.
