# Supplementary material for: Cardiogenic shock in Taiwan from 2003 to 2017 (CSiT-15 study)
Source: Crit Care. 2021 Nov 18;25:402. doi: 10.1186/s13054-021-03820-1 (PMC8600726; doi:10.1186/s13054-021-03820-1)
Supplement: Supplementary file 2 — Additional file 2. Disease diagnostic coding, procedure coding, and ATC code for medication. [file 13054_2021_3820_MOESM2_ESM.docx]

**Additional file 2.** Disease diagnostic coding, procedure coding, and ATC code for medication

Description of data: The relevant codes of disease diagnosis, medication use, and treatment procedure are presented in this table.

| **Diseases/Comorbidity** | **ICD-9-CM/ATC** | **ICD-10-CM** |
| --- | --- | --- |
| Cardiogenic shock | 785.51 | R570 |
| Congestive heart failure | 428 | I50 |
| Hypertension | 401–405 | I10–I13, I15 |
| Diabetes mellitus | 250 | E08–E13 |
| Peripheral arterial disease | 440.2–440.4, 443.9 | I70, I73.9 |
| Rheumatic disease | 710, 714, 725 | M05–M06, M32–M35 |
| Peptic ulcer disease | 531, 532, 533, 534 | K25–K28, K31.82, K56.6 |
| Dyslipidemia | 272 | E75, E77–E78 |
| Coronary artery disease | 413–414, 429.2 | I20, I25 |
| Myocardial infarction admission | 410 | I21–I22 |
| Chronic kidney disease | 403.01, 403.11, 403.91, 404.02–404.03, 404.12–404.13, 404.92, 404.93, 582, 583–583.2, 583.4, 583.6–583.7, 585–586, 588, V42, V45.1, V56 | E10.21, E11.21, I12–I13, N03, N05–N08, N14–N18, N25 |
| Stroke | 430–438 | G45–G46, I60–I63, I65–I69 |
| Malignancy | 140–239 | C00–C96, D00–D49, E31.22, J84.81, J91.0, K31.7, K63.5, Q85.0 |
| Chronic pulmonary disease | 490, 491, 492, 493, 494, 495, 496, 500, 501, 502, 503, 504, 505, 506.4 | J40–J45, J47, J60–J68 |
| Chronic liver disease | 571 | K70, K73–K76 |
| Atrial fibrillation | 427.3 | I48 |
| AIDS/HIV | 042 | B20 |
| Cardiac arrest | Cardiac arrest: 427.5  Cardiac complications (0–4): 668.1  Cardiac complications:997.1 Sudden cardiac arrest: V12.53 | Cardiac arrest: I46.2, I46.8, I46.9 Cardiac complications (0–4): O74.2, O89.1 Cardiac complications: I97.7, I97.8 Sudden cardiac arrest: Z86.74 |
| **Procedure** |  |  |
| Percutaneous coronary intervention | 33076A, 33077A, 33078A, 33076B, 33077B, 33078B |  |
| Coronary artery bypass graft | 68023A, 68024A, 68025A, 68023B, 68024B, 68025B |  |
| Heart transplantation | 68035A, 68035B |  |
| Intra-aortic balloon pump | 33079A, 33079B |  |
| Extracorporeal membrane oxygenation | 68036A, 68036B |  |
| Ventricular assist device | 68051B |  |
| Cardiopulmonary resuscitation | 47029C |  |
| **Medication** |  |  |
| Dopamine | C01CA04 |  |
| Norepinephrine | C01CA03 |  |
| Dobutamine | C01CA07 |  |
| Epinephrine | C01CA24 |  |
