# Supplementary material for: Cardiogenic shock in Taiwan from 2003 to 2017 (CSiT-15 study)
Source: Crit Care. 2021 Nov 18;25:402. doi: 10.1186/s13054-021-03820-1 (PMC8600726; doi:10.1186/s13054-021-03820-1)
Supplement: Supplementary file 3 — Additional file 3. Healthcare resource use stratified by different time periods. [file 13054_2021_3820_MOESM3_ESM.docx]

**Additional file 3.** Healthcare resource use stratified by different time periods

Description of data: This table details trends in medical costs, ICU stays, and hospital stays over time.

|  | 2003–2005 | 2006–2008 | 2009–2011 | 2012–2014 | 2015–2017 | *Trend p* |
| --- | --- | --- | --- | --- | --- | --- |
| Cost, point | 175 847± 256 980 | 177 342 ± 247 460 | 203 073 ± 287 433 | 214 626 ± 319 162 | 234 234 ± 346 551 | <0.0001 |
| ICU stay, days | 4.7 ± 8.7 | 4.4 ± 7.6 | 5.0 ± 8.5 | 5.2 ± 9.1 | 5.3 ± 8.9 | <0.0001 |
| Hospital stay, days | 11.0 ± 16.2 | 10.7 ± 14.8 | 11.8 ± 16.6 | 11.9 ± 16.7 | 11.4 ± 16.3 | <0.0001 |

Abbreviation: ICU: intensive care unit.
