# Supplementary figures and images for: Cardiogenic shock in Taiwan from 2003 to 2017 (CSiT-15 study)
Source: Crit Care. 2021 Nov 18;25:402. doi: 10.1186/s13054-021-03820-1 (PMC8600726; doi:10.1186/s13054-021-03820-1)

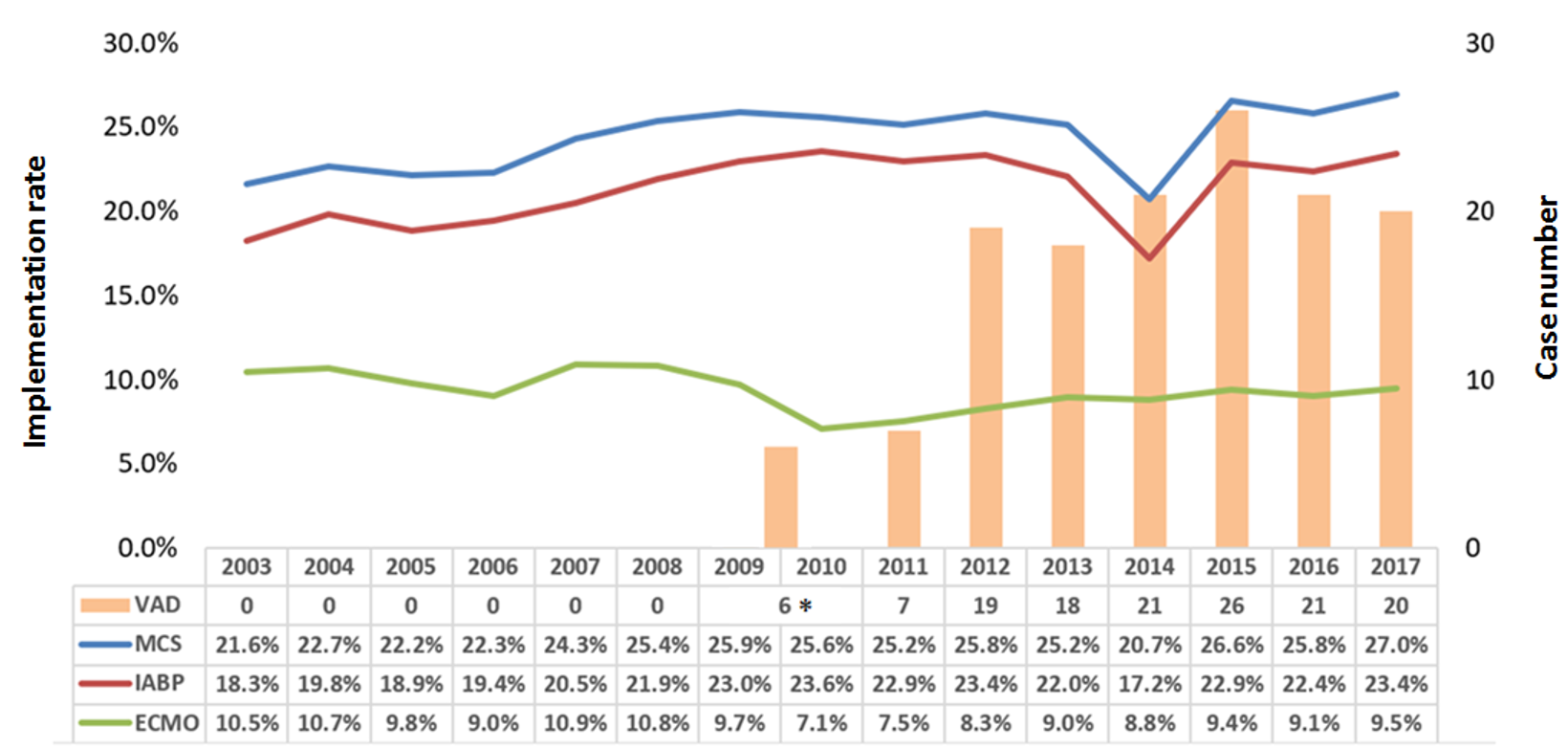

Supplement: Supplementary file 4 — Additional file 4. Annual trends of mechanicals circulatory support devices. [file 13054_2021_3820_MOESM4_ESM.tif]
