# Supplementary material for: Cardiogenic shock in Taiwan from 2003 to 2017 (CSiT-15 study)
Source: Crit Care. 2021 Nov 18;25:402. doi: 10.1186/s13054-021-03820-1 (PMC8600726; doi:10.1186/s13054-021-03820-1)
Supplement: Supplementary file 5 — Additional file 5. Baseline characteristics of patients with cardiogenic shock stratified by AMI etiology. [file 13054_2021_3820_MOESM5_ESM.docx]

**Additional file 5**. Baseline characteristics of patients with cardiogenic shock stratified by AMI etiology

Description of data: This table lists the baseline and clinical characteristics of patients according to AMI or non-AMI etiology.

|  | Non-AMI | AMI | *p* value |
| --- | --- | --- | --- |
| Overall | *N* = 38 143 | *N* = 25 906 |  |
| **Demographics** |  |  |  |
| Age (years) | 70.9 ± 15.6 | 70.2 ± 13.2 | <0.0001 |
| Male sex (%) | 21 783 (57.1%) | 17 923 (69.2%) | <0.0001 |
| **History, *n* (%)** |  |  |  |
| Congestive heart failure | 18 111 (47.5%) | 8199 (31.6%) | <0.0001 |
| Hypertension | 19 288 (50.6%) | 11 653 (45%) | <0.0001 |
| Diabetes mellitus | 13 046 (34.2%) | 9457 (36.5%) | <0.0001 |
| Peripheral arterial disease | 468 (1.2%) | 312 (1.2%) | 0.798 |
| Dyslipidemia | 4909 (12.9%) | 4695 (18.1%) | <0.0001 |
| Coronary artery disease | 14 492 (38%) | 15 468 (59.7%) | <0.0001 |
| Prior myocardial infarction | 2979 (7.8%) | 25 906 (43.2%) | <0.0001 |
| Renal failure | 3741 (9.8%) | 1820 (7%) | <0.0001 |
| Stroke | 7180 (18.8%) | 3911 (15.1%) | <0.0001 |
| Malignancy | 4533 (11.9%) | 1681 (6.5%) | <0.0001 |
| Atrial fibrillation | 7492 (19.6%) | 1998 (7.7.0%) | <0.0001 |
| **Hospital location, *n* (%)** |  |  |  |
| Northern | 17 054 (44.7) | 12 143 (46.9%) |  |
| Central | 7360 (19.3%) | 4459 (17.2%) |  |
| Southern | 5491 (14.4%) | 3933 (15.2%) |  |
| Kao-Ping | 7058 (18.7%) | 4662 (18%) |  |
| Eastern | 1180 (3.1%) | 709 (2.7%) |  |
| **Hospital level, *n* (%)** |  |  |  |
| Medical center | 12 303 (32.3%) | 10 196 (39.4%) |  |
| Regional hospital | 18 499 (48.5%) | 13 553 (52.3%) |  |
| District hospital | 7341 (19.2%) | 2157 (8.3%) |  |
| **CS conditions, *n* (%)** |  |  |  |
| Cardiac arrest | 11 729 (30.8%) | 9366 (36.2%) | <.0001 |
| STEMI | 0 | 9522 (36.8%) | <.0001 |
| NSTEMI | 0 | 16 384 (63.2%) | <.0001 |
| **Cardiology procedure, *n* (%)** |  |  |  |
| PCI | 2275 (6%) | 13 190 (50.9%) | <.0001 |
| CABG | 1168 (3.1%) | 2485 (9.6%) | <.0001 |
| Heart transplantation | 131 (0.3%) | 52 (0.2%) | 0.0009 |
| **Vasoactive agents, *n* (%)** |  |  |  |
| Dopamine | 26 621 (69.8%) | 21 534 (83.1%) | <.0001 |
| Norepinephrine | 13 196 (34.6%) | 11 621 (44.9%) | <.0001 |
| Dobutamine | 6752 (17.7%) | 5371 (20.7%) | <.0001 |
| Epinephrine | 19 381 (50.8%) | 15 425 (59.5%) | <.0001 |
| **Mechanical support** |  |  |  |
| IABP | 3421 (9%) | 10 361 (40%) | <.0001 |
| ECMO | 2695 (7.1%) | 3220 (12.4%) | <.0001 |
| VAD | 90 (0.2%) | 50 (0.2%) | 0.253 |

Abbreviations: CS: cardiogenic shock, STEMI: AMI: acute myocardial infarction, ST-segment elevation myocardial infarction, NSTEMI: non-ST-segment elevation myocardial infarction, PCI: percutaneous coronary intervention, CABG: coronary artery bypass graft, IABP: intra-aortic balloon pump, ECMO: extracorporeal membrane oxygenation, VAD: ventricular assist device.
