# Supplementary material for: Cardiogenic shock in Taiwan from 2003 to 2017 (CSiT-15 study)
Source: Crit Care. 2021 Nov 18;25:402. doi: 10.1186/s13054-021-03820-1 (PMC8600726; doi:10.1186/s13054-021-03820-1)
Supplement: Supplementary file 6 — Additional file 6. Baseline characteristics of patients with cardiogenic shock stratified by 1-year mortality. [file 13054_2021_3820_MOESM6_ESM.docx]

**Additional file 6.** Baseline characteristics of patients with cardiogenic shock stratified by 1-year mortality

Description of data: This table describes the baseline and clinical characteristics of patients in relation to 1-year mortality.

|  | Survivor | Non-survivor | *p* value |
| --- | --- | --- | --- |
| Overall | *N* = 22 596 | *N* = 41453 |  |
| **Demographics** |  |  |  |
| Age (years) | 65.5 ± 15.0 | 73.4 ± 13.9 | <0.0001 |
| Male sex (%) | 14787 (65.4%) | 24919 (60.1%) | <0.0001 |
| **History, *n* (%)** |  |  |  |
| Congestive heart failure | 8313 (36.8%) | 17 997 (43.4%) | <0.0001 |
| Hypertension | 10 721 (47.4%) | 20 220 (48.8%) | 0.0013 |
| Diabetes mellitus | 7137 (31.6%) | 15 366 (37.1%) | <0.0001 |
| Peripheral arterial disease | 219 (1%) | 561 (1.4%) | <0.0001 |
| Dyslipidemia | 4858 (21.5%) | 4746 (11.4%) | <0.0001 |
| Coronary artery disease | 12 761 (56.5%) | 17 199 (41.5%) | <0.0001 |
| Prior myocardial infarction | 2861 (12.7%) | 5278 (12.7%) | 0.797 |
| Renal failure | 1283 (5.7%) | 4278 (10.3%) | <0.0001 |
| Stroke | 2941 (13%) | 8150 (19.7%) | <0.0001 |
| Malignancy | 1455 (6.4%) | 4759 (11.5%) | <0.0001 |
| Atrial fibrillation | 3575 (15.8%) | 5915 (14.3%) | <0.0001 |
| **Hospital level, *n* (%)** |  |  |  |
| Medical center | 8270 (36.6%) | 14 229 (34.3%) |  |
| Regional hospital | 11 725 (51.9%) | 20 327 (49%) |  |
| District hospital | 2601 (11.5%) | 6897 (16.6%) |  |
| **CS conditions, *n* (%)** |  |  |  |
| Cardiac arrest | 2447 (10.8%) | 18 648 (45%) | <.0001 |
| STEMI | 4712 (20.9%) | 4810 (11.6%) | <.0001 |
| NSTEMI | 4859 (21.5%) | 11 525 (27.8%) | <.0001 |
| **Cardiac procedure, *n* (%)** |  |  |  |
| PCI | 8038 (35.6%) | 7427 (17.9%) | <.0001 |
| CABG | 1715 (7.6%) | 1938 (4.7%) | <.0001 |
| Heart transplantation | 127 (0.6%) | 56 (0.1%) | <.0001 |
| **Vasoactive agents, *n* (%)** |  |  |  |
| Dopamine | 15 442 (68.3%) | 32 713 (78.9%) | <.0001 |
| Norepinephrine | 5131 (22.7%) | 19 686 (47.5%) | <.0001 |
| Dobutamine | 3895 (17.2%) | 8228 (19.8%) | <.0001 |
| Epinephrine | 5772 (25.5%) | 29 034 (70%) | <.0001 |
| **Mechanical support** |  |  |  |
| IABP | 5655 (25%) | 8127 (19.6%) | <.0001 |
| ECMO | 1814 (8%) | 4101 (9.9%) | <.0001 |
| VAD | 58 (0.3%) | 82 (0.2%) | 0.127 |

Abbreviations: CS: cardiogenic shock, STEMI: ST-segment elevation myocardial infarction, NSTEMI: non-ST-segment elevation myocardial infarction, PCI: percutaneous coronary intervention, CABG: coronary artery bypass graft, IABP: intra-aortic balloon pump, ECMO: extracorporeal membrane oxygenation, VAD: ventricular assist device.
